# Firearm screening and secure storage counseling among home visiting providers: a cross-sectional study of SafeCare^®^ providers in the U.S.

**DOI:** 10.3389/fpubh.2024.1324656

**Published:** 2024-03-07

**Authors:** Melissa C. Osborne, Kate Guastaferro, Shelden Banks, Hari Vedantam, Shannon Self-Brown

**Affiliations:** ^1^Wellstar School of Nursing, Wellstar College of Health and Human Services, Kennesaw State University, Kennesaw, GA, United States; ^2^Department of Social and Behavioral Sciences, School of Global Public Health, New York University, New York, NY, United States; ^3^Department of Social Work and Human Services, Wellstar College of Health and Human Services, Kennesaw State University, Kennesaw, GA, United States; ^4^Department of Psychological Science, Norman J. Radow College of Humanities and Social Sciences, Kennesaw State University, Kennesaw, GA, United States; ^5^Department of Health Policy and Behavioral Sciences, School of Public Health, Georgia State University, Atlanta, GA, United States; ^6^National SafeCare Training and Research Center, Mark Chaffin Center for Healthy Development, School of Public Health, Georgia State University, Atlanta, GA, United States

**Keywords:** home visiting, firearms, parenting, firearm safe storage, injury prevention, secure storage

## Abstract

**Background:**

Firearms used in pediatric firearm deaths are most often obtained from the child’s home, making secure firearm storage initiatives imperative in prevention efforts. Evidence-based home visiting (EBHV) programs are implemented with over 277,000 families annually, providing an opportunity for secure firearm storage counseling. The purpose of this study was to assess EBHV providers’ experiences with firearm screening (“assessment”), secure storage counseling, and their perceptions for related training needs.

**Methods:**

Providers in the U.S. from SafeCare®, an EBHV program often implemented with families experiencing increased risk of child neglect and physical or emotional abuse, were invited to participate in a survey to examine firearm assessment and attitudes toward and experiences with firearm safety counseling. Survey items were primarily Likert scale ratings to indicate level of agreement, with some open-ended follow-up questions. Descriptive statistics (i.e., frequencies and percentages) were used to report item-level agreement. A *post hoc* analysis was conducted using Spearman correlation to examine the association between assessment and counseling and provider-level factors.

**Results:**

Sixty-three SafeCare providers consented to and completed the survey items. Almost three-quarters (74.6%) agreed/strongly agreed that they assess in-home firearm availability. However, 66.7% agreed/strongly agreed that they have not been adequately trained to discuss firearm safety topics. A substantial proportion (80.6%) indicated they would counsel more if materials and training on this topic were available. Response variability emerged by level of urbanicity. A *post hoc* analysis found that providers’ self-reported frequency of assessment and counseling were associated with their comfort level discussing firearm safety and whether or not they had worked with families impacted by firearm injury.

**Conclusion:**

SafeCare providers report a need for materials and training on secure firearm storage, and a willingness to provide more counseling with proper training to the families they serve. Findings illuminate the need for secure storage initiatives for EBHV programs, which have broad service reach to a substantial number of at-risk U.S. families annually.

## Introduction

Firearms have surpassed motor vehicles as the leading cause of death among children in the U.S. with >2,500 deaths recorded and > 130,000 years of potential life lost in 2021 (1). Nearly 40% of all children in the U.S. live in a home with a firearm – a number that could be an underestimate considering a surge in firearm sales in response to the COVID-19 pandemic and other civil unrest in 2020 (2). Children as young as 3-years old have demonstrated the strength needed to pull a firearm trigger (3). Additionally, among 10-to 14-year-olds, suicide rates have more than doubled since 2006 (1), and the risk of suicide increases in the presence of highly lethal means, such as firearms (4). Child maltreatment victimization increases the risk of both firearm exposure and suicidality. Over 600,000 children were victims of substantiated child maltreatment in 2021, and an even greater number are estimated to have experienced maltreatment based on self-reported data (5, 6). Childhood physical and emotional abuse is associated with increased likelihood of firearm availability, thus increasing the risk of injury associated with living with a home with a firearm (7). Additionally, experiences of child maltreatment are a risk factor for suicidality (8, 9).

Though firearm storage encompasses a spectrum of behaviors that vary in risk, the most widely accepted and comprehensive definition of secure storage is when a firearm is stored unloaded, locked up, with ammunition locked separately (10). Secure firearm storage is estimated to prevent up to 32% of pediatric firearm-related deaths due to unintentional injury and suicide (11). However, only 44% of U.S. households report keeping all of their firearms unloaded and locked (2). Thus, prevention strategies directed toward parents or other caregivers of children (hereafter referred to as “parents”), especially among families experiencing cumulative risk factors for child maltreatment, to promote secure firearm storage will have a strong impact on pediatric firearm fatalities and should be a focus of prevention efforts.

Many parents who interact with child welfare due to increased risk for reports of child maltreatment or incidents of child maltreatment are referred to evidence-based home visiting (EBHV) programs that deliver educational and supportive services in the home setting, addressing issues from prenatal care to parenting practices to home safety. Home visiting allows for more personalized interventions and involvement of the whole family, and it eliminates common service barriers such as the need for transportation and childcare. There are a multitude of positive outcomes associated with parents’ participation in EBHV programs, including reduced risk of future reports to child welfare, reduced parental depression, increased positive parenting skills, and improved child cognitive outcomes (12–16). Federal funding is dedicated to supporting EBHV programs in the U.S. through the Maternal, Infant, and Early Childhood Home Visiting (MIECHV) program. MIECHV-funded programs served over 277,000 families in 2021 (17). Providers of EBHV programs are uniquely situated to both identify and respond to safety concerns in the home as they build a strong rapport with families, assess for strengths and risks firsthand, and tailor resources to meet the unique needs of each family they serve. This is especially important for secure firearm storage counseling because attitudes toward firearms may be deeply ingrained within families and vary across factors such as urbanicity (18). Home visitors have a wide range of educational backgrounds across professional fields which may or may not prepare them to discuss secure firearm storage counseling with families.

Prior studies have examined secure firearm storage counseling practices among pediatricians and social workers with results showing overall low rates of counseling and need for training across professions (19, 20). However, more research is necessary to better understand the frequency and opportunities for these messages to reach families, to ultimately contribute to a consistent message from child-and family-serving professionals regarding secure firearm storage. EBHV providers could contribute to this messaging. However, there is currently no formal guidance or curricula for firearm secure storage counseling specific to EBHV providers. Additionally, to-date, no published studies have examined secure firearm storage counseling practices of EBHV providers. EBHV providers have a wide range of educational backgrounds from paraprofessional training to undergraduate and graduate degrees in human services. The current educational and training requirements may not adequately prepare them to discuss culturally and politically charged topics, such as firearms, with the families they serve, who often have a variety of daily life challenges and may live in homes or communities in which there is exposure to violence. Thus, the aims of this study were to (1) examine the attitudes toward and experiences with firearm screening and secure storage counseling among EBHV providers and (2) to assess differences in these findings by level of urbanicity for the setting where the provider serves families.

## Methods

### Study design

This study used a cross-sectional design, involving an online survey, administered via Qualtrics. Providers of the EBHV program, SafeCare® were invited to respond to questions about several emerging topics in home visiting. SafeCare is an EBHV for caregivers of children ages 0- to 5-years old that, as of 2023, is implemented in 27 U.S. states and 8 countries outside the U.S. The curriculum consists of three modules (i.e., parent–child interaction, home safety, and child health), each delivered in six sessions (18 total sessions). The opportunity to participate in the online survey was offered to all certified SafeCare providers in the U.S. as of September 2019 (*N* = 1,148) via email with an anonymous link to the Qualtrics survey. The survey was open for 2 weeks total, and a reminder email was sent after 1 week. Survey topics included child nutrition (21), firearm safety, and recreational marijuana use among caregivers. The current study presents results related to the firearm section. While 1,148 providers were on the National SafeCare Training and Research Center’s (NSTRC) certified provider list and would have received an email invitation to the survey, some providers may not have been actively employed at their agency at the time of distribution. For example, they may have left the organization in which they became SafeCare certified without updating their email address with NSTRC. Provider turnover is a challenge in EBHV (22, 23). It was not possible to follow up with non-responders, and, due to volume, we did not track emails that bounced back. For this reason, the precise denominator for response rate is difficult to define. The study was determined to be exempt from review by the Georgia State University Institutional Review Board.

### Measures

#### Participant demographics

All participants were asked basic demographic questions including age, gender (“male,” “female,” “transgender,” or “other [please specify]”), U.S. state, and urbanicity where they serve families. Urbanicity was captured as a mutually exclusive item with the following categories: rural (less than 2,500 people), urban cluster/suburban (2,500–50,000 people), or urban (50,000 people or more). These categories were based on the 2010 U.S. Census urban and rural classifications (24). Respondents self-reported their service area’s level of urbanicity (rural, suburban, or urban).

#### Firearm-related items

Survey questions were adapted from a prior study examining factors associated with firearm assessment and secure storage counseling among social workers in a range of practice settings (20). The survey consisted of 20-items about attitudes, knowledge, and behaviors related to firearm assessment and secure storage counseling, considering the past 2 years of service. Survey respondents were asked to rate items on a 4-point Likert scale ranging from 1 (“Strongly Disagree”) to 4 (“Strongly Agree”). For example, “I routinely assess if the parents I work with own and have access to guns;” “The families I work with are safer with a gun in the home;” and “There are more important topics to discuss than firearm safety.” Comfort level discussing firearm safety was assessed with the item, “I am generally uncomfortable bringing up firearm safety with the parents I work with and/or their families.” Binary variables were created to indicate either agreement (i.e., “Strongly Agree” or “Agree”) or disagreement (i.e., “Strongly Disagree” or “Disagree.”)

An additional five questions about firearm-related training or experiences were also part of the survey (20), including the following: (1) growing up with firearm (s) in the home (i.e., “Did your own parent or another household member ever have a gun when you were growing up?”), (2) training on firearm safety counseling (i.e., “Where have you received formal training/education in counseling clients about firearm safety?”), and (3) two questions asking the approximate number of families they have served in which fatal and non-fatal firearm injuries occurred. Participants were instructed to consider all of their years of experience when responding to these questions. Participants who indicated that a parent or other household member had a gun when they were growing up were asked an additional question regarding how that experience influenced their firearm safety counseling practices. Having grown up with a firearm was modeled as a binary variable (yes or no; note: a data point from one respondent who selected “Do not know” for this question was considered missing). Participants were provided with seven response options for the training question, including “I’ve never received training,” a list of potential training resources (e.g., local police department or 4H), and an option to indicate “Other” and specify the training resource. If participants selected “Other” and specified that they were trained in firearm safety counseling in SafeCare training, they were considered to not have any formal training. The research team members with detailed knowledge of SafeCare training did not find the content delivered in SafeCare training to be “formal training/education in counseling clients about firearm safety.” Additionally, the current study is interested in participants’ formal training aside from SafeCare. That is, we were most interested in identifying participants who had formal training outside of SafeCare in order to get a sense of the extent of firearm training among providers. Finally, regarding the experience of having served a family in which a firearm fatal or nonfatal injury occurred, a single binary variable was created to indicate if any injury or fatality had occurred (1) or if none had occurred (0).

### Analytic plan

Descriptive statistics were calculated, and frequencies and percentages were reported separately for each of the firearm-related items. Group differences were assessed by level of urbanicity, and chi-square or Fisher’s exact tests were conducted to examine differences by group. Missing data were handled via list-wise deletion. Data were analyzed using SAS 9.4 (25). Results of inferential tests were considered statistically significant for *p*-values less than.05.

### *Post hoc* analysis

Following analysis of the primary research questions, we conducted a *post hoc* analysis to explore potential correlates related to participants’ self-reported firearm assessment and secure storage counseling. Specifically, we examined the correlations between assessment and counseling and the following variables: (1) comfort level discussing firearm safety, (2) having worked with a parent or child who had a firearm injury, (3) having grown up with a firearm in the home, and (4) having had training on firearm safety counseling. To preserve the variability in response to the Likert scale items, assessment, counseling, and comfort level were modeled as ordinal-level variables, with higher scores indicating stronger agreement that participants assess for firearms, counsel for firearm safety, and feel uncomfortable discussing firearm safety with families. Having known a family with a firearm injury, having grown up with a firearm in the home, and having had training in firearm safety counseling were all binary variables, reflecting either having had (1) or not having had (0) the experience. Spearman correlations were calculated due to the ordinal nature of the data.

## Results

### Participant background

**Figure 1:**
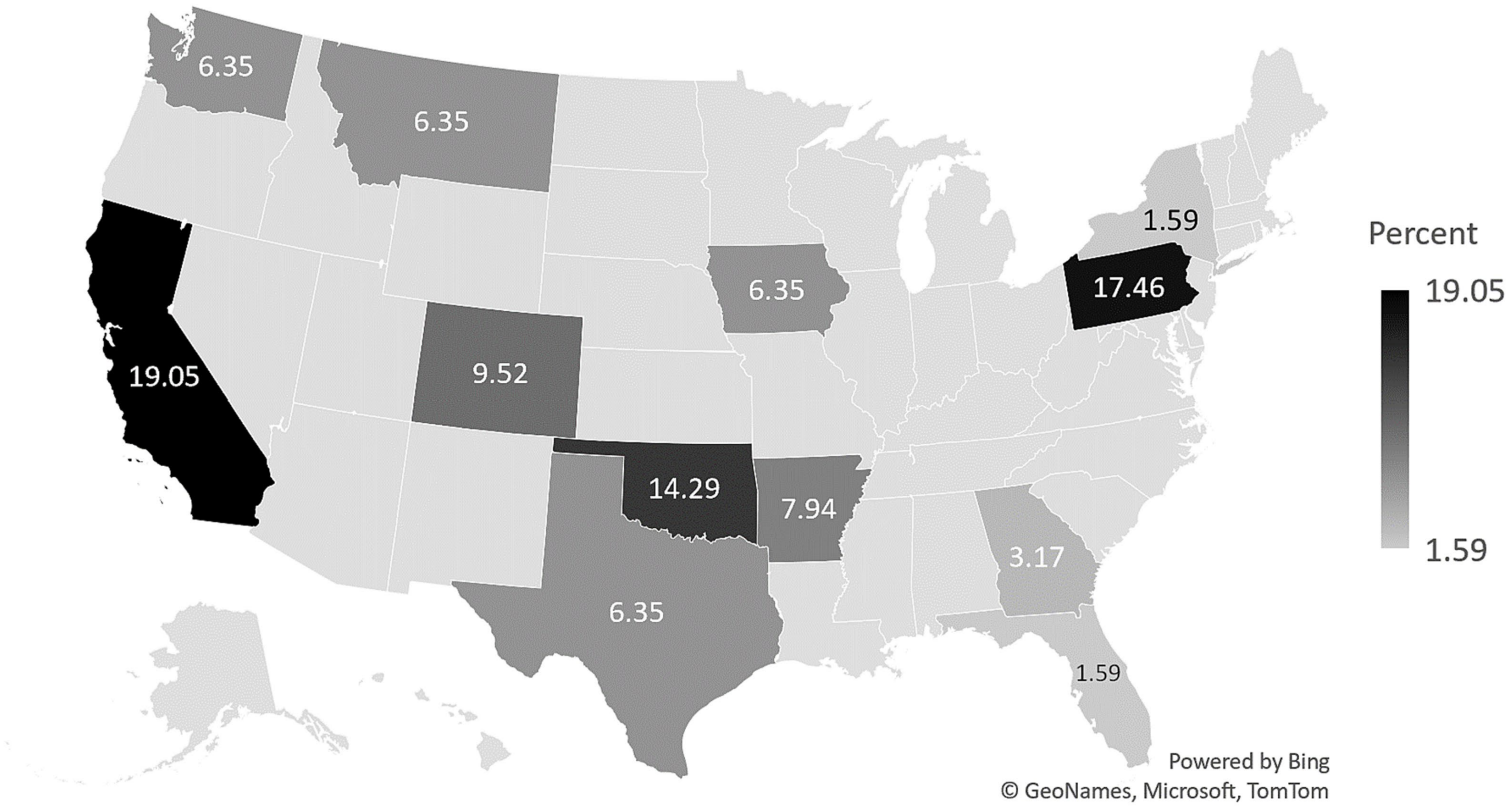
Geographic distribution of survey participants (*N* = 63).

A total of 77 SafeCare providers consented to participate in the survey, and 63 providers completed the firearm survey section. Results are presented for these 63 participants. Survey participants were geographically distributed across 12 U.S. states (see Figure 1). The sample identified predominately as female (*n* = 56; 88.89%), with 6 identifying as male (9.52%), and 1 identifying as genderqueer (1.59%). Data on age were available for 51 providers; the average age was 40 years (*SD* = 13). With regard to urbanicity of the providers’ service area was highest in the suburban areas (*n* = 32; 50.79%). The remaining providers were distributed between urban areas (*n* = 19; 30.16%) and rural areas (*n* = 12; 19.05%).

Of the 63 providers who completed the firearm survey section, 28.57% (*n* = 18) reported they had worked with at least 1 family in which a gun-related injury had occurred. Two-thirds (*n* = 40) of the providers grew up in homes with guns. Of those, 42.50% (*n* = 17) said that they would be more likely to counsel because of this history, and 47.50% (*n* = 19) said that they are more comfortable talking about gun safety with families because of this. Approximately three-fourths (*n* = 45; 73.77%) of the sample reported no “formal training” or “education” in counseling clients about firearm safety. The remaining 26.23% of those who did report training received from various sources such as an employer (*n* = 8), local police departments (*n* = 6), in college (*n* = 3), or in gun safety or hunter safety classes (*n* = 2).

### Provider attitudes toward and experiences with firearm safety counseling

Overall, 74.61% of participants agreed or strongly agreed that they routinely assess for firearms. Two-thirds, reported routinely counseling the parents they work with about firearm safety. Two-thirds of providers agreed or strongly agreed that they had not been adequately trained to discuss firearm safety. An overwhelming majority reported that they would counsel more families about firearm safety if given educational material at their agency (80.64%) and that proper training would give them credibility with parents (72.59%). Finally, in the overall sample, 21.31% of participants, agreed or strongly agreed that families would be safer with a gun in the home. See Table 1 for more details.

**Table 1:** U.S. SafeCare providers’ experiences with and attitudes toward firearm assessment and safety counseling (*N* = 63).

Item	Agreement, *n* (%)
I think counseling clients on firearm safety would be effective in reducing firearm-related injury, death, and suicide among the parents I work with and the children in their care.	51 (82.26)
I would counsel more families about firearm safety if handouts and educational material were available at my agency.	50 (80.64)
I think firearm violence has become a major public health issue.	47 (77.05)
I routinely assess if the parents I work with own and have access to guns.	47 (74.61)
Proper training on firearm safety would give me credibility with the parents I work with.	45 (72.59)
I routinely counsel the parents I work with about firearm safety.	42 (66.67)
I have not been adequately trained to discuss firearm safety.	42 (66.67)
I am likely to support gun control legislation.	33 (55.00)
There are more important topics to discuss than firearm safety.	26 (41.93)
I do not think the parents I work with would be truthful about their gun ownership and access.	26 (41.27)
The media’s coverage of gun-related issues has motivated me to counsel more about firearm safety.	25 (39.68)
I do not think my advice will change my clients’ and/or their families’ behavior regarding firearm safety.	24 (38.10)
The parents I see and the children in their care are not at risk for using a firearm to harm someone.	23 (36.51)
The parents I see and the children in their care are not at risk for firearm injury.	20 (31.74)
The families I work with are safer with a gun in the home.	13 (21.31)
It is not the responsibility of program providers to talk about firearm safety with parents.	14 (22.22)
I am generally uncomfortable bringing up firearm safety with the parents I work with and/or their families.	11 (17.46)
I am not aware of the suicide, homicide, and injury risks associated with having a firearm in the home.	10 (16.13)
I’m concerned that I will offend the parents I work with and/or their families if I talk about firearm safety.	10 (15.87)
I do not have enough time during sessions to counsel the parents I work with about firearm safety.	9 (14.28)

Agreement indicates that participants agreed or strongly agreed with the item.

### Differences in attitudes and experiences by urbanicity

**Figure 2:**
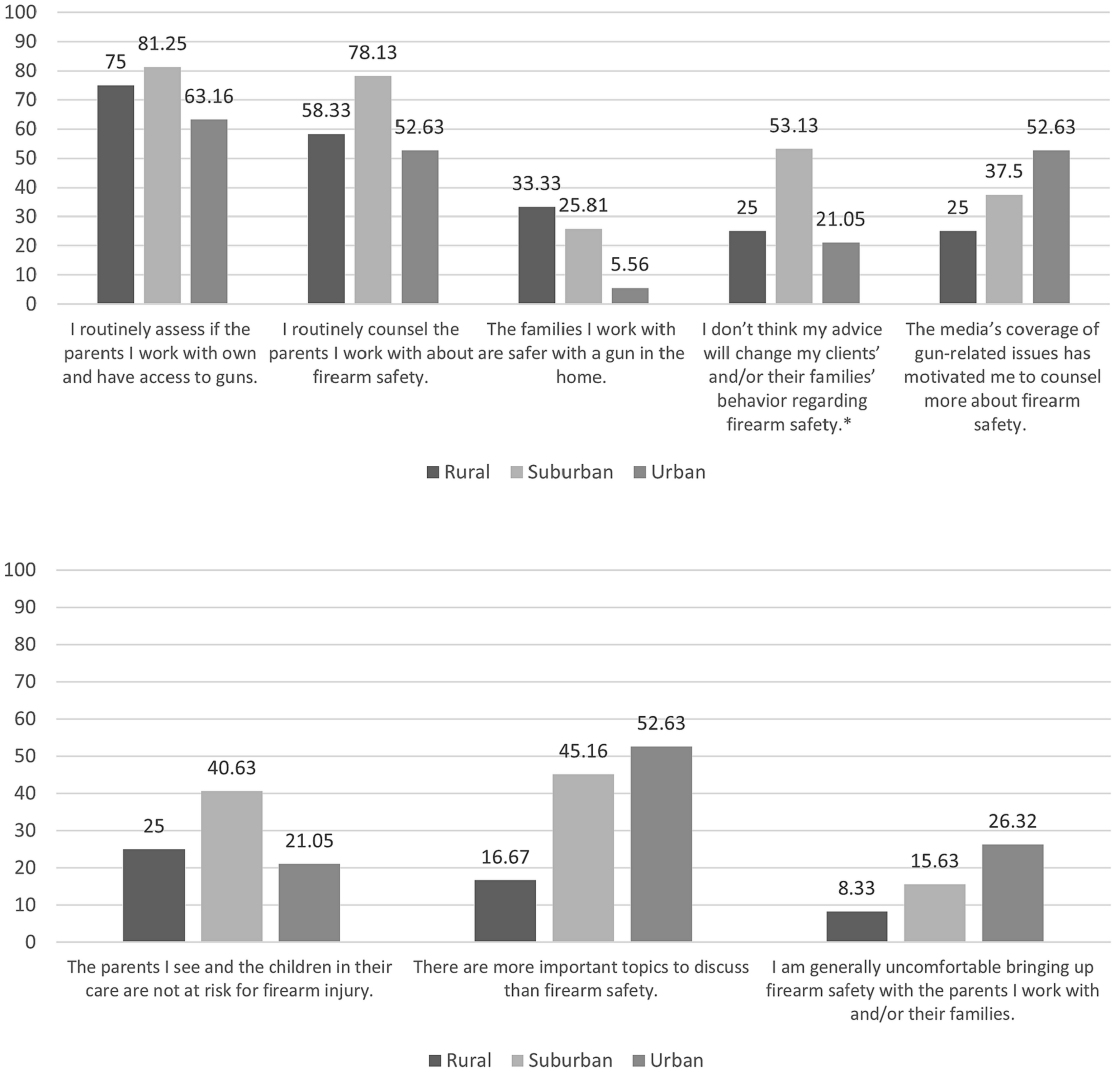
Agreement with survey items by urbanicity. ^*^p < 0.05.

Survey item results were examined by the self-reported urbanicity of the providers’ service area (rural, suburban, or urban). Assessment of firearms in the home ranged from 63.16% (*n* = 12) among providers in urban areas to 81.25% (*n* = 26) of providers in suburban areas; counseling ranged from 52.63% (*n* = 10) of providers in urban areas to 78.13% (*n* = 25) of providers in suburban areas. Chi-square test results indicated statistically significant differences by urbanicity for agreement on the following survey item: “I do not think my advice will change my clients’ and/or their families’ behavior regarding firearm safety,” *χ*^2^ (2, *N* = 63) = 6.28, *p* = 0.04. Approximately one-quarter of participants from urban (*n* = 4; 21.05%) and rural (*n* = 3; 25.00%) service areas agreed or strongly agreed with this statement. However, over half of providers from suburban areas (*n* = 17; 53.13%) reported agreement. While there were no other statistically significant differences by level of urbanicity, descriptive differences were observed for several items. For instance, one-third of providers in rural settings (*n* = 41) believe that families they work with are safer with a gun in the home; results from providers in urban environments were distinctly different, with only 5.56% (*n* = 1) of providers agreeing with this statement. Approximately half of all (*n* = 10; 52.63) providers in urban service areas surveyed believed that there were more important topics to discuss than firearm safety, while the proportion for providers in rural areas was considerably lower (*n* = 2; 16.67%). See Figure 2 for more details.

### *Post hoc* analysis

There was a moderate, statistically significant, inverse correlation between participant comfort level with discussing firearm safety and both assessment (*r* = −0.46, *p* < 0.001) and counseling (*r* = −0.34, *p* = 0.006). On average, higher levels of discomfort were associated with lower levels of assessment and counseling. There was a moderate, statistically significant, positive correlation between having worked with a family with a firearm injury and assessment (*r* = 0.34, *p* = 0.007) and counseling (*r* = 0.30, *p* = 0.02). Working with such a family was associated with higher levels of assessment and counseling. There were no statistically significant correlations between having grown up with a firearm or having been trained in firearm safety counseling and self-reported assessment and counseling with families in the past 2 years. See Table 2 for details.

**Table 2:** Correlation matrix for firearm assessment, secure storage counseling, and related background factors.

	Assess	Counsel	Comfort level	Family injury	Grew up w/FA	Training status
Assess^a^	1.00					
Counsel^a^	0.56*	1.00				
Comfort Level^a^	−0.46*	−0.34*	1.00			
Family Injury^b^	0.34*	0.30*	−0.20	1.00		
Grew up w/FA^b^	0.12	0.003	0.01	0.06	1.00	
Training Status^b^	−0.12	0.02	−0.10	−0.23	0.03	1.00

Spearman correlation coefficients presented; Assess = “I routinely assess…;” Counsel = “I routinely counsel….;” Comfort Level = “I am generally uncomfortable bringing up firearm safety with parents….”; Family Injury = At least 1 parent or child the respondent worked with had been injured by a firearm; Grew up w/FA = Growing up, respondent’s own parent or other household member had a gun; Training Status = Respondent reported receiving “formal training/education in counseling clients about firearm safety”.

^a^1 = Strongly disagree to 4 = Strongly agree.

^b^1 = yes, 0 = no.

*p < 0.05.

## Discussion

This study examined EBHV providers’ experiences with and attitudes toward firearm assessment and secure firearm storage counseling. We also explored associations between four provider-level factors and firearm assessment and secure storage counseling experience. Our findings indicate that almost 75% of SafeCare providers report assessing families they serve for firearm availability, and 67% report counseling families in firearm safety. Three-quarters of providers reporting firearm assessment is substantial and is comparable to or higher than rates reported by social workers (20) and pediatricians (19, 26). This may be due to the sample consisting of SafeCare-trained providers. SafeCare is the only EBHV program with a dedicated home safety module (27) and assessing the home for safety hazards is part of that module. Yet, 67% of the providers surveyed report that they have not been adequately trained to discuss firearm safety. Additionally, over 80% said they would counsel more families on secure firearm storage if given the proper materials and training. This presents an opportunity for researchers and community and professional organizations with expertise in secure firearm storage counseling for parents to work with EBHV program developers and purveyors to incorporate such content into their programs. This has the potential to reach hundreds of thousands of families with young children in the U.S.

Importantly, there was variability in provider survey responses by urbanicity, by comfort level, and by proximity to firearm injury. Notably, a substantially greater proportion of providers from rural areas indicated that families are safer with a gun in the home than urban providers. This is likely due to differences in gun culture between rural and urban areas. Research has found regional variation in firearm ownership in samples from both the general population (28, 29) and those at risk for child welfare involvement (7). Additionally, self-reported social gun culture has been linked to gun ownership (30), and gun culture, which also varies regionally, may contribute to social norms surrounding the use of firearms for self-protection (31, 32). When developing secure firearm storage initiatives for EBHV programs, it may be important to tailor the focus of the messaging by urbanicity or by reason for firearm ownership, addressing both provider pre-existing barriers to counseling on secure storage and parent barriers to secure storage.

Findings also indicated that providers who reported greater comfort discussing firearm safety with parents were more likely to engage in firearm assessment and firearm safety counseling with families they serve. Increasing EBHV provider’s likelihood of firearm assessment and secure storage counseling could begin with increasing their self-efficacy and comfort around these activities. This could easily be incorporated into EBHV workshop training, to include modeling conversations with parents around firearms and firearm storage and allowing home visitors to practice and receive feedback. Prior research has shown that training that includes vignettes, discussion, and suggestions for integration into practice has been found effective in increasing self-efficacy in secure storage counseling among medical students (33). This is in line with principles of social learning theory (34) that are used in training healthcare professionals (35). It could be applied to training EBHV providers in secure firearm storage counseling as well.

Finally, EBHV provider’s experience of serving a family in which a firearm injury had occurred was associated with firearm assessment and safety counseling. It may be the case that a provider knowing a family that has experienced a firearm injury especially motivates them to engage in prevention efforts and opens up a natural opportunity for discussions and training on secure firearm storage; however, there is a lack of literature to confirm this assertion. Familiarity with a family impacted by firearm injury may be akin to hearing narratives or, “illustrative examples of others’ experiences” (36, 37). No known research has examined the impact of narratives on provider behaviors; although experts in environmental health have noted the importance of including narratives in communication with healthcare providers to increase their engagement on this topic (38). Narratives have been found to impact behavior change at the patient level in health topic areas aside from firearm injury prevention, for example, youth substance use and hypertension (39, 40). However, the literature on narratives is mixed, as some studies have found that narratives have no effect on patient behavior (36). More research is needed to clarify how the impact of serving families with firearm injuries may drive the behavior of EBHV providers.

Related, the End Family Fire! Campaign, a firearm injury prevention campaign, prominently features “safe stories,” a collection of stories of people who came close but did not die by firearm suicide because of secure firearm storage (41). Given the relationship between providers’ exposure to family experiences of firearm injuries and their assessment and counseling practices, EBHV providers’ likelihood of assessing for firearms and counseling on secure firearm storage could be increased by including such success stories in training. In the future, these training strategies should be developed and rigorously tested. Ultimately, more research on the use of narratives is needed in terms of their impact on provider secure firearm storage counseling and on parent storage behavior.

While not the focus of the current study, participants were also asked about the feasibility of firearm safety education as part of EBHV, in terms of the time they have with families for delivering this content. Specifically, participants were asked to rate the item, “I do not have enough time during sessions to counsel the parents I work with about firearm safety.” on a scale from Strongly Disagree to Strongly Agree. Less than 15% of participants agreed with this statement, indicating that the vast majority of participants felt that firearm safety is a topic they have time to discuss. This is notable, because other professionals who deliver secure firearm storage counseling to parents, such as pediatricians, report the lack of time to devote to this issue as a primary barrier to implementation (19, 42). While EBHV providers work with a much smaller segment of the population than pediatricians do, children in the families receiving home visiting services may be at greater risk for firearm-related outcomes, based on their possible exposure to factors such as child maltreatment (4, 7, 8). EBHV providers also spend more time with the families they serve than other professionals, as services are commonly structured around weekly visits that span the course of months or years (17). Thus, EBHV providers are an important part of the collective response of child-and family-serving professionals to firearm injury prevention.

Findings from this study should be interpreted with some important limitations in mind. First, study participants were recruited through convenience sampling from a single EBHV program, SafeCare. This limits the generalizability of the conclusions, and future work should employ more representative samples. While many EBHV programs cover home safety topics on some level, SafeCare is the only EBHV program with a dedicated core module on home safety. Thus, this sample of providers may be stronger in discussing home safety topics with parents compared with EBHV providers who have not been trained to deliver SafeCare. Although, despite the training on home safety, two-thirds of providers reported feeling inadequately trained to discuss firearm safety. Future research should incorporate more rigorous sampling methods and expand recruitment to more EBHV program providers. However, some providers are trained to deliver multiple home visiting programs (43), and study participants were asked to reflect on all families for whom they delivered services in the last 2 years, not only SafeCare families. Thus, there may have been study participants who deliver SafeCare as well as other home visiting programs and who were considering families participating in programs other than SafeCare.

Additionally, this study is also subject to selection bias. It could be the case that providers who are more open to emerging topics in home visiting may also be more likely to participate in a survey about emerging topics, and those who are resistant to discussing challenging or controversial topics with parents are less likely to respond to such a survey. Also, while we invited all certified SafeCare providers in the U.S. to join the study, the primary firearm items asked participants to reflect on the past 2 years of service. Thus, some participants may have been new to service delivery and would have been reflecting on a time period of less than 2 years. This also means they would not have had as many opportunities to discuss firearm safety with families as more experienced participants had. Also, there was a low response rate to the survey, impacting generalizability of the findings. We are unable to define a denominator for response rate calculation due to in ability to track providers who may have no longer been employed with their agency but were still included in SafeCare records. Using the number of providers on the email list as a denominator (*N* = 1,148) as the most conservative approach, the response rate was 6.7%. However, it is important to note that numerous U.S. states and regions were represented by the respondents. Future work should include more rigorous sampling methods to improve generalizability to the broader population of EBHV providers. Finally, the use of the term “firearm safety” in the survey, as opposed to “secure firearm storage” or “safe storage” may have impacted the way the participants interpreted the items using that term, as firearm safety could encompass more than just secure storage. The survey used in this study was previously implemented with social workers (20) and items were altered only with regard to home visiting-specific terminology to maintain consistency. Future research would benefit from using more direct and commonly-used terminology.

## Conclusion

This study examined EBHV providers’ attitudes toward and experiences with firearm assessment and secure storage counseling, using a sample of SafeCare providers in the U.S. Three-fourths of SafeCare providers reported assessing for firearms in the home, and two-thirds reported counseling on firearm safety. This is not surprising given SafeCare’s dedicated module on home safety. However, two-thirds of providers indicated that they were not adequately trained to discuss firearms, and over three-fourths said they would counsel more families if given the proper materials and training. This points to a training need for the EBHV workforce. EBHV providers are an important part of the response to pediatric firearm injury prevention, and more research is needed to develop and test strategies that prepare providers to discuss firearms with the families they serve.

## Data availability statement

The datasets presented in this article are not readily available because the data supporting the findings of this study are available upon request from the authors. Requests to access the datasets should be directed to MO, mcowart3@kennesaw.edu.

## Ethics statement

The studies involving humans were approved by Georgia State University Institutional Review Board. The studies were conducted in accordance with the local legislation and institutional requirements. The ethics committee/institutional review board waived the requirement of written informed consent for participation from the participants or the participants’ legal guardians/next of kin because the researchers did not interact with participants directly, and no identifying information was collected in the online survey.

## Author contributions

MO: Conceptualization, Formal analysis, Methodology, Software, Supervision, Writing – original draft, Writing – review & editing. KG: Writing – review & editing, Conceptualization. SB: Writing – original draft, Writing – review & editing. HV: Visualization, Writing – original draft, Writing – review & editing. SS-B: Conceptualization, Resources, Supervision, Writing – review & editing.
